# Clinical Experience With Combined Pantoprazole and Pheniramine in the Treatment of Persistent Hiccups: A Case Series

**DOI:** 10.7759/cureus.107185

**Published:** 2026-04-16

**Authors:** Yusuf S Sahin

**Affiliations:** 1 Internal Medicine, Araban İlçe Devlet Hastanesi, Gaziantep, TUR

**Keywords:** case series, intractable hiccups, pantoprazole, pheniramine, singultus

## Abstract

Hiccups (singultus) are generally benign and self-limiting conditions; however, in some patients, they may become persistent and require treatment. Although proton pump inhibitors (PPIs), chlorpromazine, and various centrally acting agents are commonly used, evidence for alternative treatment options remains limited. While PPIs are frequently utilized for hiccups that develop secondary to dyspepsia, there is limited data in the literature to support antihistamines as an effective treatment modality, and clinical data supporting their use are also limited. This case series aimed to evaluate the clinical utility and safety of the combination of intravenous pantoprazole and pheniramine, an H1 antihistamine with anticholinergic properties, in the treatment of persistent hiccups.

## Introduction

Hiccups (singultus) are sudden, involuntary contractions of the diaphragm and inspiratory muscles followed by reflexive glottis closure. While usually benign, hiccups lasting over 48 hours are classified as “persistent,” and those exceeding one month as “intractable” [1,2]. Although the exact global incidence remains poorly defined, persistent hiccups account for thousands of emergency department visits and outpatient consultations annually. These conditions often signal serious underlying disorders and significantly impair quality of life by causing exhaustion, malnutrition, and sleep disturbances [3]. The pathogenesis involves a complex reflex arc, comprising afferent, central, and efferent limbs, that is regulated by neurotransmitters such as dopamine, acetylcholine, and glutamate [4,5]. In adults, predisposing factors typically include advanced age, male gender, and comorbidities such as cerebrovascular accidents or gastrointestinal irritation [1,6].

Despite the availability of established treatments such as chlorpromazine, metoclopramide, or baclofen, their clinical success is inconsistent. Furthermore, their use is frequently limited by adverse effects like sedation and extrapyramidal symptoms, particularly in elderly populations [3]. Following an unexpected clinical success in an elderly patient with multiple comorbidities, I investigated a novel therapeutic approach targeting both the vagal afferent triggers and the central neurotransmitter pathways of the hiccup reflex. This study presents the clinical experience of combined intravenous or oral pantoprazole and pheniramine across diverse clinical profiles, proposing this combination as a safer and potentially synergistic alternative for managing persistent hiccups.

## Case presentation

Case 1 

A 49-year-old male presented to the outpatient clinic with a one-week history of persistent hiccups. The patient's medical history was significant only for chronic dyspepsia. Physical examination and vital signs were within normal limits. Laboratory investigations were unremarkable. The patient was administered an intravenous (IV) infusion of 40 mg pantoprazole and 45.5 mg pheniramine in 100 mL of normal saline over 15 minutes. Following the infusion, the hiccups completely resolved; however, symptoms recurred approximately 60 minutes later. Subsequently, a maintenance regimen of oral pantoprazole (40 mg/day) and oral pheniramine (22.7 mg/day) was prescribed. At the two-week follow-up visit, the patient reported complete resolution of symptoms with no further recurrence.

Case 2

A 28-year-old male presented to the clinic with a three-day history of intermittent, persistent hiccups, occasionally subsiding for 15-minute intervals. The patient's past medical history was unremarkable. Physical examination and vital signs were within normal limits. Laboratory investigations - including complete blood count, liver and kidney function tests, electrolytes, glucose, HbA1c, vitamin B12, ferritin, folate, and viral serology - were within normal ranges, except for a low 25-hydroxyvitamin D level (17.19 ng/mL; reference range: 30-100 ng/mL).

Given that the patient was fasting during Ramadan, dyspepsia was suspected as a trigger. Consequently, an initial bolus of 40 mg IV pantoprazole was administered. Observation over the following 30 minutes showed no clinical improvement. Subsequently, an IV infusion of 45.5 mg pheniramine in 100 mL of normal saline was administered over 15 minutes. The hiccups ceased completely three minutes after the completion of the pheniramine infusion. The patient was discharged with a regimen of oral pantoprazole (40 mg/day) and oral pheniramine (22.7 mg/day). At the two-week follow-up visit, it was confirmed that the symptoms had not recurred.

Case 3

An 80-year-old male presented to the clinic with a five-day history of persistent hiccups, primarily occurring during the evening hours. The patient's past medical history was unremarkable. Physical examination and vital signs were within normal limits. Laboratory results for complete blood count, liver and kidney function, electrolytes, glucose, and bilirubin levels were normal. Before this visit, the patient had been treated with oral chlorpromazine in an outpatient setting; however, his relatives had discontinued the medication after two days due to adverse effects, including blunted affect, excessive sedation, and altered mental status.

As the patient was not experiencing active hiccups at the time of admission, an oral regimen of pantoprazole (40 mg/day) and pheniramine (22.7 mg/day) was initiated. One week later, a scheduled telephone follow-up consultation with his relatives revealed that there had been no improvement in his symptoms. This case represents the only instance in this series where a patient failed to benefit from the oral pantoprazole and pheniramine combination after failing previous chlorpromazine therapy.

Table 1 summarizes the demographics, clinical presentations, diagnostic findings, treatment regimens, and follow-up outcomes of the three presented cases.

**Table 1:** Summary of patient characteristics, interventions, and clinical outcomes IV: intravenous

Patient	Age in years/sex	Clinical presentation (duration)	Past medical history	Diagnostic findings	Treatment regimen	Clinical outcome and follow-up
Case 1	49/male	Persistent hiccups (1 week)	Chronic dyspepsia	Unremarkable	Initial: IV pantoprazole (40 mg) + IV pheniramine (45.5 mg). Maintenance: oral pantoprazole (40 mg/day) + pheniramine (22.7 mg/day)	Initial resolution with recurrence at 60 minutes. Sustained complete remission on oral maintenance at the 2-week follow-up
Case 2	28/male	Intermittent persistent hiccups (3 days)	Unremarkable	Unremarkable except for low 25-hydroxyvitamin D (17.19 ng/mL)	Initial: IV pantoprazole bolus (40 mg) followed 30 minutes later by IV pheniramine (45.5 mg). Maintenance: oral pantoprazole (40 mg/day) + pheniramine (22.7 mg/day)	Hiccups ceased completely 3 minutes post-pheniramine infusion. Sustained complete remission at the 2-week follow-up
Case 3	80/male	Persistent hiccups (5 days)	Unremarkable (prior treatment failure with chlorpromazine)	Unremarkable	Initial and maintenance: oral pantoprazole (40 mg/day) + oral pheniramine (22.7 mg/day)	No clinical response. Hiccups persisted at the 1-week follow-up

## Discussion

In this case series, the clinical response and safety of a combination of intravenous and oral pantoprazole with pheniramine were evaluated in three patients of different age groups suffering from persistent hiccups. My findings suggest that this combination may serve as a potential therapeutic option, particularly in clinical scenarios where conventional treatments are contraindicated or associated with a high risk of adverse effects. The most frequently utilized agents in the literature for persistent hiccups include proton pump inhibitors, dopamine antagonists (for example, chlorpromazine, metoclopramide), and GABAergic agents [1,3]. However, some patients remain refractory to these interventions, or their use is limited by adverse effects [2]. Treatment selection becomes increasingly complex in elderly patients and those vulnerable to polypharmacy.

Regarding antihistamines, there is limited data in the literature to support their use as an effective treatment modality. Pheniramine, a classic H1 antihistamine, possesses significant sedative and anticholinergic effects on the central nervous system [7]. It is particularly noteworthy because of its potential to modulate multiple components of the hiccup reflex arc [6,7]. Through its anticholinergic properties, it may suppress acetylcholine-mediated transmission involved in phrenic nerve-mediated muscular activity [6]. Additionally, its central sedative effects may reduce the excitability of the hiccup center in the medulla oblongata [5,7].

Analysis of these cases highlights a rapid clinical response following intravenous administration. In the second case, the lack of response to pantoprazole monotherapy, followed by the cessation of hiccups three minutes after the completion of the pheniramine infusion, suggests a possible synergistic interaction. However, given the short timeframe, a delayed therapeutic effect from the initial pantoprazole bolus cannot be entirely excluded, though the close temporal association with pheniramine administration is highly notable. Similarly, the first case demonstrated an initial response to intravenous treatment with sustained remission achieved through oral maintenance. Conversely, the lack of response to oral therapy in the third case underscores that this combination is not a consistently effective regimen. This therapeutic failure could be attributed to age-related factors, the specific etiology of the hiccups, pharmacokinetic variables, or differences in central sensitivity rather than a predictable response. Moreover, variations in bioavailability and onset of action between oral and intravenous routes likely contributed to the observed clinical outcomes.

A key clinical implication of this series is that pheniramine may offer a more favorable safety profile in patient groups where other options are limited, such as benzodiazepines due to respiratory depression risks, or dopamine antagonists like chlorpromazine due to extrapyramidal side effects and tolerability issues.

This study has several limitations, including a small sample size, the lack of a randomized controlled design, and limited follow-up duration. Further investigations, such as upper gastrointestinal endoscopy and neuroimaging, to elucidate the underlying etiology, were not performed in any of the cases. The potential for a placebo effect, spontaneous resolution, and the heterogeneity of underlying etiologies must also be considered.

## Conclusions

This case series demonstrates that the combination of intravenous and/or oral pantoprazole and pheniramine may serve as a safe and accessible alternative for managing persistent hiccups, particularly when conventional therapies like chlorpromazine are poorly tolerated. The observed clinical response likely stems from a synergistic mechanism: pantoprazole addresses peripheral gastrointestinal triggers, while pheniramine provides central neuromodulation of the hiccup reflex arc through its anticholinergic properties. While the successful resolution of symptoms in two of these cases is encouraging, these findings represent an off-label use based on clinical observation. Further prospective, randomized controlled trials are needed to establish standardized dosing regimens and confirm the overall clinical effectiveness of this therapeutic approach.
